# Epidemiology and Traits of Mobile Colistin Resistance (*mcr*) Gene-Bearing Organisms from Horses

**DOI:** 10.3390/microorganisms10081499

**Published:** 2022-07-25

**Authors:** Madubuike Umunna Anyanwu, Ishmael Festus Jaja, Obichukwu Chisom Nwobi, Anthony Christian Mgbeahuruike, Chinaza Nnenna Ikpendu, Nnenna Audrey Okafor, James Wabwire Oguttu

**Affiliations:** 1 Microbiology Unit, Department of Veterinary Pathology and Microbiology, University of Nigeria, Nsukka 400001, Nigeria; anthony.mgbeahuruike@unn.edu.ng; 2 Department of Agriculture and Animal Health, Florida Campus, University of South Africa, Johannesburg 1709, South Africa; joguttu@unisa.ac.za; 3 Department of Veterinary Public Health and Preventive Medicine, University of Nigeria, Nsukka 400001, Nigeria; obichukwu.nwobi@unn.edu.ng; 4 Department of Veterinary Microbiology, Michael Okpara University of Agriculture, Umudike 440101, Nigeria; ikpendu.chinaza@mouau.edu.ng; 5 Department of Microbiology, University of Nigeria, Nsukka 400001, Nigeria; nnenna.okafor.pg04212@unn.edu.ng

**Keywords:** antimicrobial resistance, colistin resistance, equine, mobile colistin resistance (*mcr*) gene

## Abstract

Mobile colistin resistance (*mcr*) genes (*mcr*-1 to *mcr*-10) threaten the efficacy of colistin (COL), a polymyxin antibiotic that is used as a last-line agent for the treatment of deadly infections caused by multidrug-resistant and extensively drug-resistant bacteria in humans and animals. COL has been used for more than 60 years for the prophylactic control and treatment of infections in livestock husbandry but not in horses. Polymyxin B is used for the prophylactic control and empirical treatment of infections in horses without conducting sensitivity tests. The lack of sensitivity testing exerts selection pressure for the acquisition of the *mcr* gene. By horizontal transfer, *mcr*-1, *mcr*-5, and *mcr*-9 have disseminated among horse populations globally and are harbored by *Escherichia coli*, *Klebsiella*, *Enterobacter*, *Citrobacter*, and *Salmonella* species. Conjugative plasmids, insertion sequences, and transposons are the backbone of *mcr* genes in the isolates, which co-express genes conferring multi- to extensive-drug resistance, including genes encoding extended-spectrum β-lactamase, ampicillinase C, fosfomycin, and fluoroquinolone resistance, and virulence genes. The transmission of *mcr* genes to/among bacterial strains of equine origin is non-clonal. Contact with horses, horse manure, feed/drinking water, farmers, farmers’ clothing/farm equipment, the consumption of contaminated horse meat and its associated products, and the trading of horses, horse meat, and their associated products are routes for the transmission of *mcr*-gene-bearing bacteria in, to, and from the equine industry.

## 1. Introduction

Antimicrobial resistance (AMR), especially in Gram-negative bacilli (GNB) that are resistant to virtually all available antimicrobial agents, continues to be a growing global public and animal health threat, impacting negatively on the socioeconomic development of nations irrespective of their income level. Terrifyingly, 127 million human deaths were directly attributed to resistant infections in 2019 [1]. There is no need to reiterate the outrageous economic impact that AMRs have on national, regional, and global economies [1,2,3,4,5,6]. Chromosome-borne and non-conjugative plasmid-encoded resistance genes are mutationally acquired and vertically transferred to bacterial progenies within a clone, and thus by their very nature are self-limiting. However, conjugative plasmid-encoded resistance genes rapidly spread horizontally/laterally among bacterial populations without clonal restriction, leading to the rapid loss of the therapeutic/clinical efficacy of antimicrobial agents [7,8,9]. Plasmids are self-replicating extrachromosomal genetic materials in bacteria that drive acquired resistance. They are mobile genetic elements (MGEs) with the capacity to carry other genetic elements, such as insertion sequences, transposons, integrons, and gene cassettes that readily capture/transfer genes from/to other organisms by mechanisms of horizontal gene transfer (HGT), namely conjugation, transduction, and transformation [8,9,10]. Indeed, plasmid movement between different bacterial species and lineages represents a major source of AMR [8,9].

Colistin (COL), a polycationic peptide belonging to the polymyxin class of antibiotics (including polymyxin B), is one of the few last-line antibiotics used for the treatment of deadly infections caused by multidrug- and extensively drug-resistant bacteria, including carbapenem-resistant strains of bacteria in humans and animals [11,12,13,14]. COL was largely abandoned soon after its production due to its nephrotoxic and neurotoxic potential, which is related to its dosage and pharmacokinetics, alongside the availability of other effective and safe antibiotics [11,15,16]. However, with restrictions in a few developed countries, COL has been used for more than six decades in livestock for growth promotion and the prophylactic control and treatment of infections caused by GNB. In humans, COL has been used mostly for the treatment of topical infections and cystic fibrosis, and for selective decontamination of the gut [11,15]. The emergence and rapid spread of carbapenem-resistant organisms forced clinicians to resort to COL for human therapeutic management [15,16]. In 2015, it was discovered that a plasmid-encoded COL determinant (the mobile COL resistance (*mcr*-1) gene) emerged, threatening the clinical utility of COL [17]. Currently, ten *mcr* genes (*mcr*-1 to *mcr*-10), with many variants and subvariants, have been detected in isolates/samples from humans, animals, and the environment in more than 65 countries on all seven continents [12,18,19]. The *mcr* genes encode transmembrane enzymes, including phosphoethanolamine (pEtN) transferase, which mediates COL resistance by attaching a pEtN moiety to the lipopolysaccharide (LPS) of lipid A in the outer membrane of Gram-negative bacilli, thereby eliminating the negative charge on the LPS to which cationic COL/polymyxins have an affinity [20]. There has been extensive discourse on the methods used in the detection of these genes and their specific characteristics [14,21,22,23,24,25]. Although the transmission of chromosome-borne and non-conjugative plasmid-encoded *mcr* genes is clonally restricted [7,11], conjugative plasmid-borne *mcr* genes are rapidly transferred to other organisms (i.e., inter/intraspecies and genera transmission) as these plasmids are highly promiscuous, thus jeopardizing antimicrobial/COL therapy [7,11,25]. Therefore, there is a need to understand the magnitude, genetic context, and epidemiological relationships of *mcr*-gene-bearing organisms that have been isolated from different ecological niches. Reports on *mcr*-gene-mediated COL resistance in animals, especially in food-producing animals (FPAs), are more abundant than those in humans and the environment [13,16,20,23,26,27,28,29,30,31], indicating that animals are a huge reservoir for plasmid-mediated COL resistance (PMCR) and warrants increased surveillance of animal sources as part of a process to reduce the spread of COL resistance. However, horses appear to be an underestimated reservoir of *mcr*-gene-bearing organisms.

Horses are highly prized animals that are used for various purposes in different parts of the world, including sports (such as polo games), transportation (as a beast of burden), festivals, security (as mountain troops), pets and companions, and as a source of meat and other products such as hide [32,33,34]. The annual economic impact of the global equine industry is estimated at $300 billion dollars, creating employment for about 1.6 million people, and generating a gross domestic product of over $100 billion in North America alone [35]. Annually, the size of the horseback-riding industry is rapidly increasing, and the estimated number of horse riders is congruently increasing worldwide [36,37]. Thus, there is an increased chance of transmission of zoonotic resistant pathogens from horses to humans and vice versa due to increased contact with horses [36]. Therefore, it is important to evaluate this risk due to this potential public health threat. Antimicrobials, including critically important ones such as polymyxin B and ceftiofur, are used for the treatment of infections and prophylaxis in horses, but the latter mode of use (prophylactic control) is widely criticized [38,39,40]. Globally, empirical treatment (without conducting the sensitivity test) for horses is allowed [41,42,43]. This practice potentially exerts selection pressure, stimulating the development of AMR and the acquisition of plasmid-encoded transmissible *mcr* genes in exposed bacteria. Most worrisome is the acquisition/co-expression of *mcr* genes with plasmid-encoded genes, conferring resistance to other high-priority and highest priority last-resort antimicrobials, such as extended-spectrum cephalosporins (mediated by the production of extended-spectrum β-lactamases (ESBL) and plasmidic Ampicillinase C (pAmpC)), carbapenems (mediated by carbapenemase production), glycylines (mediated by the production of tetracycline/tigecycline destructatses/flavin monooxygenases and resistance-nodulation-division efflux pumps), and fosfomycin (mediated by the production of glutathione S transferases) [13,44]. The potential for companion animals, including horses, as reservoirs for ESBL- and carbapenemase-producing organisms has been extensively reviewed [42,43,44,45,46], as have *mcr* genes in dogs and cats [27,45]. Information on COL resistance in horses would be helpful in selecting therapy based on local sensitivity data for treatment in equine practice and to curb the spread of *mcr*-gene-bearing bacteria (MGBB). Moreover, understanding the epidemiology and traits of *mcr*-bearing bacteria from horses regarding phenotypic resistance, the genetic context of the *mcr* gene, population structure, and genotypic features creates the impetus to tackle the problem of AMR [13]. This mini-review discusses the potential sources, transmission routes, impacts, occurrence, and phenotypic and genotypic traits of *mcr*-bearing isolates from horses.

## 2. Possible Sources, Transmission Routes, and Impacts of *mcr*-Gene-Bearing Organisms in Horses

Horses are herbivores; they only feed on plant material. Thus, forages contaminated with COL-r organisms, originating from human or animal ejections/manure and even biocides that led to the development of AMR, constitute a route for the colonization of horses by these organisms [16,46]. Rainfall run-off from an environment contaminated with anthropogenic/agricultural wastes, such as sewage and wastewaters from homes, hospitals, laboratories, pharmaceutical industries, livestock farms, slaughterhouses, and aquafarms, potentially carry COL-r organisms to vegetations/grasslands [13,47,48]. Wild animals, such as birds, flies, and rodents, that often interact with anthropogenic/agricultural wastes can acquire MGBB from these wastes and deposit them on forages or stored feed (hay)/feedstuff used to supplement the diets of horses [13]. Access to drinking water that is sourced from the surface, or underground water contaminated by anthropogenic/agricultural wastes (sewage and wastewater from homes, hospitals, pharmaceutical industries, laboratories, and livestock farms) could result in the colonization of animals by COL-r organisms [47,48,49]. Individuals (such as horse groomers, hoof-farriers, jockeys, owners, and caretakers) with poor hand hygiene may directly contact horses and potentially contaminate forages, feed, or gears (straps, brushes, saddles, etc., used in riding/restraining horse mouths) with COL-r organisms.

In most countries, antibiotics belonging to several classes, including polypeptides (polymyxin B), macrolides, aminoglycosides, ansamycins, penicillin, cephalosporins, quinolones, sulphonamides, tetracyclines, trimethoprim, and metronidazoles, are used for the prophylactic control and empirical treatment of bacterial infections without conducting sensitivity tests, especially in situations where mortality rates are high, e.g., sepsis in foals [40,41,43,50]. Polymyxin B is used for the management of endotoxemia in horses and, at an anti-endotoxic dose (which is lower than the antimicrobial dose), for the treatment of systemic inflammatory response syndrome (SIRS) [40,51]. The use of polymyxin B in subtherapeutic concentrations potentially exerts selection pressure for the development of AMR. However, the use of the highest priority CIAs (HP-CIAs) without a sensitivity test in horses is not allowed in some countries, such as Sweden [40]. The use of antibiotics in undiagnosed or misdiagnosed cases and for an unrecommended duration in horses also leads to the development of AMR [40]. In most developing countries, antimicrobial use is not strictly controlled, and non-professionals/animal owners use critically important drugs without the supervision of a veterinarian [34]. Thus, in these nations, it is possible to manage infections in horses with COL. The administration of COL by untrained individuals potentially results in the development of COL resistance. Veterinary healthcare workers (VHWs) are known to have been colonized by COL-r and MGBB [52], and the role of the veterinary hospital environment as a reservoir for COL-r organisms is recognized [53]. Therefore, the nosocomial infection of horses by resistant organisms could occur following visitation to veterinary hospitals through the hands of unhygienic VHWs, horse caretakers, and/or the hospital environment [54]. The crowding of horses during shows, races, and sales is a putative risk for the exchange of resistant organisms between animals. The exchange of organisms could occur through the inhalation of respiratory droplets since these organisms have been isolated from the respiratory tract (trachea and lungs) of horses [55,56].

A horse infected/colonized by COL-r organisms is a putative source for spreading these organisms to the community through individuals who make direct and indirect contact with them [54,57]. The trade of racehorses is a potential route for the dissemination of MGBB from one place to another [13,58]. The cross-contamination of horse meat by COL-r organisms during slaughter, especially in unhygienic slaughterhouses, is a risk for further transmission of MGBB through the local or international horse meat trade [16,26,34,59]. Slaughterhouse personnel handling horse carcasses/meat, especially those with poor infection prevention control (such as poor hand hygiene and personal hygiene) and those who fail to wear personal protective equipment, are at risk of infection by COL-r organisms [26]. These individuals serve as vehicles, spreading the microorganisms to the public [26]. In developing countries, slaughterhouse wastes, such as the internal organs of slaughtered animals, constitute an important source of animal protein for dogs, and horse meat is a potential source of protein in pet foods. The handling and consumption of contaminated raw and undercooked horse meat by humans and possibly carnivores (following the consumption of horse meat that may be used in pet foods) is also a putative risk for the acquisition of MGBB [16,59,60].

**Figure 1:**
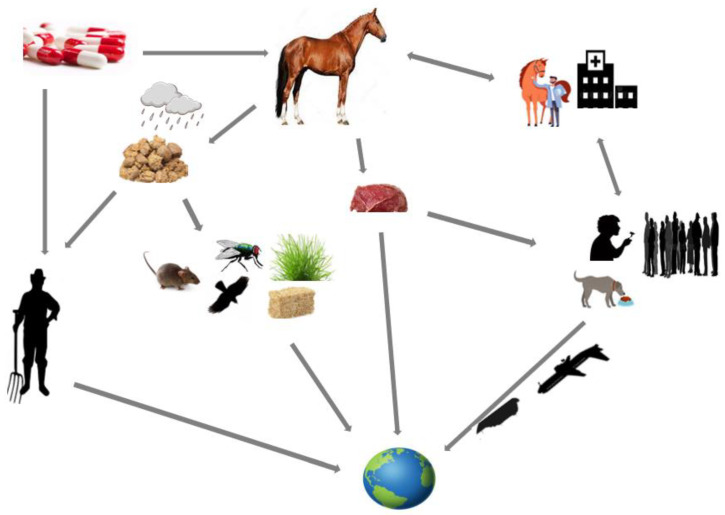
Possible sources of *mcr*-gene-bearing organisms in horses and their routes of transmission in the One Health triad.

The rearing of horses generates huge amounts of forage residuals and horse manure. Stalling a horse for a month could create approximately one ton of stall residuals [35]. The inadequate/improper disposal of stall residuals/horse manure into the environment constitutes a potential health risk [49]. Flies that feed on/breed in animal manure could carry MGBB from the manure and may transport it to humans and animals by contaminating food, water, and wounds [13]. The discharge of COL-r organisms through feces by horses on fields during horse races and the use of insufficiently composted/anaerobically digested horse manure as organic fertilizer in crop farms are potential means through which these organisms spread to the environment, potentially contaminating soil and plants [34]. Rainfall runoff may carry these organisms from manure in the environment/on farmland to other places, contaminating water bodies that may be sources of sustenance to other organisms (such as habitats for reptiles/aquatic animals and drinking water for free-range animals) [13,48]. The groomers, caretakers, riders, and jockeys of horses colonized by COL-r organisms could easily become infected from contaminated horse gears/bodies, especially following direct contact with the oral/anal region of the animal [34]. These individuals may subsequently transfer these organisms to their households and to the public [61,61]. Through local and international travel, colonized individuals may transport MGBB from horses to other locations, thus increasing the spread of these organisms [62]. Indeed, MGBB in the equine industry has one health ramification: they may disseminate to human environmental settings through diverse routes (Figure 1). Unfortunately, the treatment of infections associated with MGBB is often difficult or impossible due to limited therapy options; thus, such infections are usually fatal.

## 3. Epidemiology and Traits of *mcr*-Gene-Bearing Bacteria Isolated from Horses in Different World Regions

### 3.1. Africa

An ESBL-producing and COL-r ST405 *E. coli* that was isolated between 2014–2015 from horse manure in Algeria harbored transferrable *mcr*-1 [57], suggesting that a pandemic high-risk extraintestinal pathogenic *E. coli* (HiR-ExPEC) clone [63] has been present among the equine population in Africa since 2014 at least. Horse manure is a potential source for the dissemination of MGBB into human and environmental settings following human–animal contact with horse manure and the improper disposal of untreated/inadequately treated manure, respectively. The organism also co-expressed *bla*_TEM-12_ encoding resistance to β-lactam antibiotics, suggesting that COL selection pressure could have emanated from the use of other antimicrobials, especially β-lactams. The presence of ST405 *mcr*-1-positive *E. coli* in horses is of public health concern because this *E. coli* clone is associated with the global dissemination of ESBL genes [16]. ST405 *mcr*-1-positive *E. coli* is widely spread throughout human–animal–environment ecosystems in Algeria, has been detected in bovine manure [57] and in a wild monkey [64], and is associated with infertility and urinary tract infections in humans [65,66]. COL is used as a metaphylactic treatment and feed additive to promote growth in the Algerian livestock industry [67]. Thus, there is a possibility that horses acquired the organism from persons such as groomers, caretakers, and jockeys, or through the consumption of herbage/drinking water contaminated by anthropogenic/agricultural wastes. Furthermore, it is possible that garbage flies picked up the organisms from another place and deposited them on horse manure during feeding/breeding.

### 3.2. Europe

The *mcr*-9 gene was detected in various plasmids, particularly in IncHI2 and IncHI2A (Table 1), in 30 clinical ESBL-producing strains (in 15 *Enterobacter cloacae* complex, 10 *E. coli*, 4 *Klebsiella oxytoca*, and 1 *Citrobacter freundii*) recovered from various clinical samples from horses in Sweden [56], suggesting that various promiscuous plasmids evolved *mcr*-9 in diverse *Enterobacterales* that were circulating among the equine population in the Nordic region of Europe and that the IncHI2 plasmid is the commonest MGE driving *mcr*-9 among the equine population in Europe. This finding also suggests that *Enterobacter* is the major trafficker of *mcr*-9 in horses. IncHI2 has been reported as the predominant plasmid spreading *mcr*-9 [68], and *Enterobacter* species (spp.) have been associated with *mcr*-9 dissemination in humans, animals, and the environment more than any other organism [69,70,71,72,73]. The *mcr*-9-bearing isolates were extensively diverse, belonging to different STs (*E. coli*, six STs; *Enterobacter* spp., seven STs; and *Klebsiella* spp., three STs), including HiR pathogenic epidemic clones, indicating that the spread of *mcr*-9 among enterobacteria in horses is not clonally restricted. Among the STs of the isolates, *E. coli* ST1861 is associated with carriage of the ESBL gene in horses in the Netherlands [54]. The isolates co-expressed 24 additional resistance genes (including ESBL—*bla*_SHV-12_, pAmpC—*bla*_CMY-82_, fosfomycin—*fosA*, and PMQR—*qnrA1*, *qnrB2*, and *qnrB10* genes) conferring resistance to nine antimicrobial classes (Table 1). This suggests that horses in Europe are reservoirs for organisms harboring a cocktail of multiple resistant genes with the potential to cause difficult-to-treat diseases in humans and animals. Even more worrisome is that these genes encode resistance to cephalosporins and fluoroquinolones that are recommended as off-label options for equine medicine in Sweden [40], suggesting that the use of these drugs has exerted selection pressure and has prompted acquisition of the *mcr*-9 gene by the strains. COL is used in humans and during the postweaning time on pig farms in Sweden [74], suggesting that these settings may be sources for the emergence of *mcr*-9-bearing organisms. However, none of the *mcr*-9-bearing strains possessed *qseB*-/*qseC*-like genes, which are the regulatory genes that are speculated to be responsible for inducing the acquisition of *mcr*-9 and the expression of COL resistance [75,75]. This implies that *mcr*-9 might not actually be induced, as has been speculated, and warrants further investigation. Furthermore, some of the *mcr*-9-bearing isolates exhibited levels of COL MIC that were less than the recommended joint CLSI-EUCAST Ecological cut-off value (≥2mg/L), meaning that the use of the current breakpoints as criteria for assessing isolates for *mcr* genes leads to underestimation of the magnitude of PMCR globally as *mcr*-9 is silently disseminating. This highlights that, irrespective of the COL MIC exhibited, screening of all GNB isolated from horses for *mcr* genes should be performed in order to determine the genes’ actual prevalence in the equine population. There is a need for greater use of whole genome sequencing (WGS) in the surveillance of PMCR, or at least using polymerase chain reaction methods that target all the currently known *mcr* genes (*mcr*-1 to *mcr*-10).

In the UK, *mcr*-9 and *bla*_SHV_ were detected on different plasmids in ESBL-producing *E. coli* that were isolated from several equine hospitals [40], suggesting that the genes possibly originated from different sources and that horses are sources for the dissemination of MGBB in veterinary hospitals. This also indicated that *mcr*-9 is trafficked by the diversity of *Enterobacterales* (*E. coli* and *Enterobacter* spp.) in the European equine industry. COL has been used more in patients residing in the UK than in patients residing in other European countries [77]. Therefore, COL selective pressure for the acquisition of the *mcr*-9 could have emanated from human settings and is possibly also due to the use of cephalosporins in horses. It is worth noting that neither of the two *Aeromonas* that were isolated from horses in Germany harbored *mcr*-1, *mcr*-2, or *mcr*-3 genes [78]. Furthermore, none of the ESBL-producing *E. coli* isolates from horses in France harbored *mcr*-1, *mcr*-2, or *mcr*-3 genes [79].

### 3.3. South America

An MDR and ESBL-producing ST711 *E. coli* carrying a new *mcr*-5 variant, named *mcr*-5.3 on a novel 5361 bp plasmid, was isolated from the lung tissue of a horse that never received COL and died of pneumonia in 2012 in Brazil [55], suggesting that *E. coli* that co-produces MCR-5 and ESBL has been present in South America for at least almost a decade and is associated with difficult-to-treat diseases in horses. The *mcr*-5 gene has been detected in *E. coli* isolates from poultry birds in Brazil [80]. The livestock sector could be the source of *mcr*-5 in the Brazilian equine sector since massive amounts (up to 10 g/ton of feed) of polymyxins/COL was used in livestock feed as a growth enhancer in Brazil [81]. However, this practice was banned in Brazil in November 2016 [81]. Tn3-type transposon and eight other resistance genes (including ESBL gene *bla*_CTX-M-8_), conferring resistance to three antimicrobial classes and VAGs, existed in the *E. coli* strain of equine origin (Table 1), suggesting that diverse MGEs (transposons and plasmids) drive COL resistance in the Brazilian equine industry and that horses are potential reservoirs for organisms that carry multi-resistant genes and are capable of causing hard-to-treat diseases, thus posing a risk for the health of animals and infected individuals. However, the strain remained susceptible to cefoxitin, ciprofloxacin, enrofloxacin, ertapenem, imipenem, and meropenem, suggesting that these HP-CIAs could be useful for the management of *mcr*-gene-associated infections in horses in Brazil. Moreover, the *E. coli* strain was susceptible to COL (MIC 2 mg/L), further showing that COL-susceptible isolates are evading screening for *mcr* and are silently disseminating plasmid-mediated COL resistance [13]. This further supports the need to screen isolates irrespective of the COL MIC exhibited.

### 3.4. North America

In the USA, the *mcr*-9 gene, flanked upstream and downstream by insertion sequences (ISs) IS*26* and IS*903B*, respectively, was detected on the IncHI2 plasmid in an ST45 MDR and ESBL-producing *Salmonella* Newport that was isolated from an equine veterinary clinic during an outbreak among horses during 2003–2004 [76]. This suggests that *Enterbacterales* that co-produce MCR-9 and ESBL have been circulating in North America for at least almost two decades. This also suggests that diverse genetic elements (plasmids and ISs) are involved in the capture and transfer of *mcr*-9, and further indicates that IncHI2 is the commonest plasmid disseminating *mcr*-9 in the equine sector. The isolate possessed *qseB*/*qseC* genes with *ygiW* on the chromosome, but these genes were not associated with the *mcr*-9 gene. However, *qseBC* was downstream of *mcr*-9 in the IncHI2 plasmids of some COL-susceptible (MIC ≤ 0.5 mg/L) *Salmonella enterica* Saintpaul that were isolated from retail meat samples in the US, further suggesting that the induction of *mcr*-9 by *qseBC* expression is context-dependent and differs among isolates with different strain backgrounds [82]. The USA Food and Drug Administration approved the use of COL in FPAs, but not as a growth promoter in animal feed, suggesting that the livestock industry could be a source of *mcr* genes in the equine sector in the USA [20,23]. Nonetheless, the equine *Salmonella* strain also possessed genes encoding virulence factors (*Salmonella* Pathogenicity Islands (SPI) I and II, and fimbriae for adhesion) and co-expressed 21 other resistance determinants, including genes encoding ESBL (*bla*_SHV-12_) and pAmpC (*bla*_CMY-2_), and the PMQR gene *qnrB2* (Table 1). This indicates that the isolate is highly virulent and capable of causing difficult-to-treat disease outbreaks, thus posing a threat to public health. The salmonellosis outbreak caused by this virulent strain and others resulted in the closure of a veterinary teaching hospital, affected all the institution’s missions, and had a substantial financial impact of USA $4.12 million [83]. However, the isolate remained susceptible to meropenem, imipenem, and tigecycline, highlighting the need for AST and antimicrobial stewardship in order to preserve the efficacy of these critically important last-resort agents.

## 4. Conclusions

This review showed that a diversity of organisms, such as *E. coli*, *Klebsiella* spp., *Citrobacter* spp., *Enterobacter* spp., and *Salmonella* spp., have disseminated various *mcr* genes in the equine industry (Table 1). Similar to isolates from humans, the environment, and other animal species [13,15,18,22,23,24,25,26,27,28], *E. coli* is the predominant organism spreading *mcr* genes among the horse population. Although COL use appears to be disallowed in horses (although its use is possible in countries with lax antibiotic controls), polymyxin B is used in the management of endotoxaemia and at a subtherapeutic (below antiendotoxic) concentration in horses for the treatment of SIRS. Thus, selection pressure for the acquisition of the *mcr* gene in the equine industry is exerted by the inappropriate use of polymyxin B and other antibiotics often used empirically without conducting sensitivity tests. Sources of MGBB in the equine sector include grasses/herbages and drinking water, vectors (i.e., flies), horse handlers (i.e., caretakers, groomers, and VHWs contaminated by anthropogenic/agricultural wastes), animal vectors, and contaminated fomites. Hospital visitation/admission and crowding during horse races, shows, and sales increase the risk of colonization of horses by COL-r organisms. Sanitation by the regular removal of bedding litter from horse stables, vector screening, vaccination, the use of non-antibiotic alternatives (such as probiotics, prebiotics, synbiotics, and antimicrobial peptides), prompt accurate laboratory diagnosis whenever possible, and preventing the misdiagnosis of bacterial infection by assessing procalcitonin [40,84] could help to reduce the rate of colonization of horses by COL-r organisms/MGBB. The banning of nontherapeutic uses of COL in FPAs will also influence the equine industry as it will reduce the rate of contamination of grasses/drinking water and of humans by MGBB emanating from anthropogenic/agricultural settings. The screening of archived isolates from horses by WGS is important in order to understand the magnitude and time of emergence of MGBB in the equine industry.

The conjugative plasmids of different replicons and the incompatibility, insertion sequences, and transposons drive COL resistance in the equine industry. The IncF, IncA/C, and IncHI2 plasmids seem to be the predominant plasmid types in the strains isolated from horses. Transmission of the *mcr* gene among horse strains is non-clonal and diverse highly virulent zoonotic pandemic/epidemic and commensal clones of *E. coli*, *Klebsiella* spp., *Citrobacter* spp., *Enterobacter* spp., and *Salmonella* spp. are circulating among the equine industry in these regions. Isolates from horses co-express *mcr* genes together with virulence and resistance genes, including genes conferring resistance to last-resort antimicrobials such as those encoding ESBL, pAmpC, and plasmid-mediated quinolone and fosfomycin resistance. Thus, they are potential superbugs that may cause difficult-to-treat disease outbreaks with pandemic potential in the equine population, and may potentially result in morbidities, mortality, reduced production, and huge economic losses.

Contact with horses, horse manure, flies that feed on/breed in horse manure, and horse stable workers/their clothing and equipment, are potential routes for the acquisition of COL-r organisms/MGBB. The consumption and handling of undercooked or raw horse meat and its associated products are putative routes for colonization by COL-r organisms/MGBB. Horse meat can be contaminated at the slaughterhouse, packaging, and/or selling/retail points by handlers (slaughterhouse personnel, meat sellers, or buyers/consumers) of these meats and flies in open-air markets found in developing countries. The trade of horses and horse meat and its associated products is a route for spreading MGBB from the equine sector to other places. Insufficiently treated/untreated horse manure and slaughterhouse sewage are potential sources for the dissemination of COL-r organisms/*mcr* genes into human, soil, botanical, and aquatic environments, especially when used as organic fertilizer in farmlands. Anaerobic digestion and the composting of animal manure before use as an organic fertilizer eliminates the risk of acquiring COL-r organisms/*mcr* genes from animal manure [85].

Indeed, by horizontal/lateral and vertical transfer, the *mcr*-1, *mcr*-5, and *mcr*-9 genes have disseminated in the global equine industry (Table 1). However, more studies are needed worldwide (especially in Gulf countries with a high population of horses) to estimate the prevalence of MGBB in this industry. There would be an increased transmission of MGBB in the global equine industry, alongside the consequent potential stable-to-plate and stable-to-environmental transmission of superbugs, if efforts to curtail COL resistance in the horse supply chain are not enhanced. This further highlights the need for a One Health approach.

## Figures and Tables

**Table 1:** Studies reporting on plasmid-mediated colistin resistance in horses.

Country	Source of Isolates	Date of Isolation (*mcr* Gene Assayed)	Number of Isolates Tested for *mcr*	Identified Gene/Variant (Number of Organisms)	Sequence Type and/or Phylogroup (Virulence Genes)	Plasmid (Associated Insertion Sequence)	Additional Resistance Traits	Reference
Algeria	Horse manure	2016–2018 (*mcr*-1-*mcr*-5)	-	*mcr*-1 (1 *E. coli*)	-	-	*bla*_TEM-12_	[57]
Sweden	Uterus, wound, trachea, skin biopsy, urine, and thrombophlebitis	2015–2018 (*mcr*-1 to *mcr*-10)	56	*Mcr*-9 (15 *Enterobacter cloacae* complex, 10 *E. coli*, 4 *Klebsiella oxytoca*, and 1 *Citrobacter freundii*)	*E. coli*: ST1861, ST2557, ST9329, ST1252, ST1423, and ST4398;	IncFIB, IncFII, IncFIC, IncFI, IncP, IncA/C, and Col (MG828)	*bla*_SHV-12_, *bla*_TEM-1B_, *bla*_ACT_, *bla*_CMY-82_, *bla*_OXY_, *bla*_SCO-1_, *aac*(6′)*-Ib*, *aadA2*, *ant*(3″)*-Ia*, *aph*(3′)*-Ia*, *aph*(3″)*-Ib*, *aadA1*, *catA2*, *dfrA19*, *qnrB2*, *qnrA1*, *qnrB10*, *tet*(D), *fosA*, *mdf*(A), *ere*(A), *sul1*, and *sul2*	[56]
					*Enterobacter clocae* complex: ST113, ST116, ST1021, ST51, ST102, ST88, and ST254;			
					*Klebsiella oxytoca*: ST238, ST37, and ST2;			
					*Citrobacter freundii*: ST233			
USA	Horse veterinary clinic environment	2003–2004 (*mcr*-1 to *mcr*-10)	31	*mcr*-9 (1 *Salmonella* Newport)	ST45 (SPI-I and II, *fim* and other fimbrial colonization factors)	IncHI2 (IS*26* and IS*903B*)	*bla*_CMY-2_, *bla*_SHV-12_, *bla*_TEM-1B_, *aph*(4)*-Ia*, *aac*(6’)*-Iaa*, *aadA2*, *aac*(3)*-IV*, *aph*(*6*)*-Id*, *aph*(3″)*-Ia*, *aph*(3″)*-Ib*, *mdf*(A), *catA2*, *floR*, *fexA*, *sul1*, *sul2*, *dfrA19*, *tet*(A), *tet*(D), *qnrB2*, and mutation in *parC*	[76]
Brazil	Lungs of dead horse	2012 (*mcr*-1 to *mcr*-10)	1	*mcr*-5.3 (1 *E. coli*)	ST711 (*gad*, *lpfA*, and *iss*)	-	*bla*_CTX-M-8_, *bla*_TEM-1B_, *aac*(3)*-IId*, *aadA2*, *strA*, *strB*, *sul2*, and *dfrA12*	[55]

*mcr*, mobile colistin resistance gene; -, no data; Additional resistance traits, resistance factors identified in one *mcr*-positive isolate or pooled factors in more than one *mcr*-bearing isolate; Virulence genes, genes from *mcr*-bearing *E. coli* isolates except otherwise stated; Sequence type, Warwick multilocus sequence type of *mcr*-bearing isolates; Plasmid, plasmid types identified in one or pooled *mcr*-bearing isolates; Inc., incompatibility.

## Data Availability

Not applicable.
